# Circular RNA circEPSTI1 accelerates cervical cancer progression via miR-375/409-3P/515-5p-SLC7A11 axis

**DOI:** 10.18632/aging.202518

**Published:** 2021-02-02

**Authors:** Peng Wu, Chunxiang Li, Dong mei Ye, Kenan Yu, Yuxuan Li, Hailin Tang, Gaoshen Xu, Shuijing Yi, Zhiwei Zhang

**Affiliations:** 1 Department of Critical Care Medicine, Hengyang Maternal and Child Health Hospital, Hengyang 421001, Hunan Province, China; 2 Yueyang Maternal and Child Health Hospital, Yueyang 414000, Hunan Province, China; 3 Innovative Practice Base for Postgraduate Training of Basic Medicine and Clinical Collaboration, University of South China and Yueyang Maternal and Child Health Hospital, Yueyang 414000, Hunan Province, China; 4 Key Laboratory of Cancer Cellular and Molecular Pathology in Hunan Province, Cancer Research Institute of Hengyang Medical College, University of South China, Hengyang 421001, Hunan Province, China; 5 Department of Gynecology, The Third Xiangya Hospital of Central South University, Changsha 410013, Hunan Province, China

**Keywords:** cervical cancer, circEPSTI1, circular RNAs, miR-375, SLC7A11

## Abstract

Background: Circular RNAs (circRNAs) is one kind of non-coding RNAs (ncRNAs) and exert crucial functions in biological processes and intracellular gene expression modulation. However, the biological roles and expression status of the majority of circRNAs still remain unknown in cervical cancer.

Results: In this study, circEPSTI1 (hsa_circRNA_000479) was significantly upregulated in cervical cancer. We first discovered the impact of circRNA on cell ferroptosis in cervical cancer. Interestingly, circEPSTI1 attenuates the effect of ferritin which is mediated by SLC7A11 based on lipid peroxidation measurements and reduced glutathione and glutathione (GSH/GSSG) assay.

Conclusions: circEPSTI1-miR-375/409-3P/515-5p-SLC7A11 axis affected the proliferation of cervical cancer via the competing endogenous RNAs (ceRNA) mechanism and was relative to ferroptosis. Our findings provided experimental evidences which revealed that circEPSTI1 might act as a new and useful biomarker for monitoring and treatment target for cervical cancer.

Methods: The expression of circEPSTI1 was examined in cervical cancer cells. Then, we observed the impact of circEPSTI1 expression on the proliferation of cervical cancer by loss-of-function assays both *in vivo* and *vitro*. RIP and luciferase reporter assay revealed that circEPSTI1 sponges miR-375, miR-409-3p and miR-515-5p to upregulate SLC7A11 expression. We applied mouse xenograft experiments in mice to validate our results.

## INTRODUCTION

Cervical cancer is the fourth most common malignant tumor and the fourth leading cause of cancer death in women in the worldwide. There were 311,000 deaths and 570,000 newly diagnosed cervical cancer patients all around the world in 2018, according to statistics from the International Agency for Research on Cancer (IARC), [1]. Despite various treatment methods such as radiotherapy, chemotherapy and surgery, there is no adequate and effective method to treat cervical cancer, showing a low survival rate and a poor prognosis, mainly due to relapse, metastasis and drug resistance [2, 3]. At present, the combined treatment of bevacizumab, paclitaxel and carboplatin is the standard first-line targeted therapy and chemotherapy for cervical cancer. This scheme significantly improves the survival rate compared with the use of platinum drugs alone [4]. Although in recent years some new drugs have been applied, the current therapies for advanced or relapsed cervical cancer has generally proved to be disappointing. Therefore, there is an urgent need for some new therapeutic targets, and targeted therapy has become a trend.

Circular RNAs (circRNAs) are one type of single lined non-coding RNAs transcripts, which can regulate the expression of several key genes by combining with microRNA (miRNA) or other molecules at various level [5–7]. Most circRNAs are located in the cytoplasm which have shown huge miRNA binding capabilities, and have been identified as miRNA sponges and enhancing downstream gene expression by sponging miRNA [8–11]. In recent years, researchers are increasingly concerned about the role of circRNAs in cancer, including breast cancer and cervical cancer thanks to the progress of RNA-sequencing technology and bioinformatic analysis [12, 13]. For example, CiRS-7, an early discovered circRNA, was revealed to be an ideal molecule for miR-7 sponge, containing over seventy miR-7 miRNA target sites, so it can regulate miR-7 activity on downstream mRNA [14]. Circ-ITCH sponges miR-214, miR-17 and miR-7, thus suppressing the Wnt/β-catenin pathway [15, 16]. CircSLC26A4 promotes cervical cancer progression via the miR-1287-5p/HOXA7 axis [17]. The circEPSTI1 affects the apoptosis and proliferation of breast cancer cell via the ceRNA mechanism of miR-4753/6809-BCL11A axis [18]. Though the ceRNA mechanism, the circKIF4A accelerates TNBC progression via miR-375-KIF4A axis [19]. circFBXW7 encodes the FBXW7-185aa protein and sponges miR-197-3p to upregulate FBXW7 and suppress TNBC progression [20]. Growing evidence shows that circRNAs have the potential to become a promising biomarker for diagnosis and therapeutic targets of cancer.

Ferroptosis is a new type of cell death which is not the same as autophagy, apoptosis, and necroptosis, at both the biochemical and morphological levels. It is a unique status with lipid peroxidation, resulting in reactive oxygen species (ROS) accumulation [21, 22]. Accumulated intracellular lipid hydroperoxides are converted into lipid alcohols by GSH which is commonly mediated by GPX4 and thus the ferroptosis is repressed. System xc^−^-mediated transmembrane transport of extracellular cystine plays a vital role in the formation of GSH and cysteine in cells [23, 24]. System xc^−^ is a glutamate and cystine antiporter consisting of solute carrier family 3 membrane 2 (SLC3A2) and solute carrier family 7 membrane 11 (SLC7A11). In addition, inhibiting SLC7A11-mediated cystine uptake by erastin, an inhibitor or cystine deprivation leads to lack of intracellular GSH and hence resulting in ferroptosis-mediated cell death [21, 25, 26]. As a hotspot of cancer research, ferroptosis is recognized as a promising target in cancer treatment.

In the current study, a novel circRNA was identified in cervical cancer. This new circRNA is located on chromosome 13q14 which is originated from EPSTI1, hence being named as circEPSTI1 (genomic location: chr13: 43528083-43544806; circBase: hsa_circ_0000479). Functional assays indicated that silence of circEPSTI1 inhibited the proliferation of cervical cancer cell. In addition, circEPSTI1 bound to miR-375, miR-409-3P and miR-515-5p as a miRNA binder to upregulate SLC7A11 expression. Further analysis showed that knockdown of circEPSTI1 induced ferroptosis by downregulating SLC7A11 through the mechanism of ceRNA in cervical cancer. Overall, our research indicated that circEPSTI1 inhibit ferroptosis and be utilized as potential treatment target and ideal biomarker in cervical cancer.

## RESULTS

### CircEPSTI1 is highly expressed in cervical cancer cells

**Figure 1:**
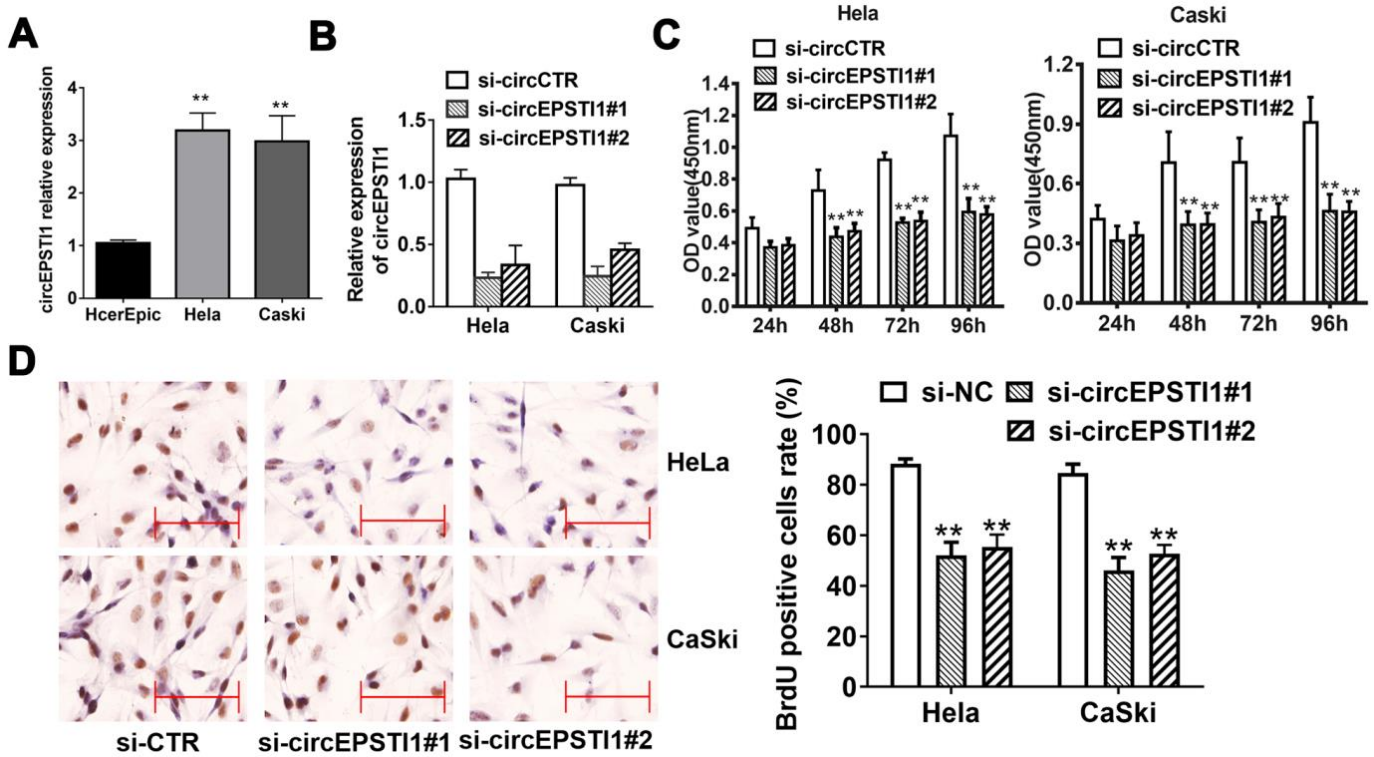
**CircEPSTI1 is highly expressed in cervical cancer cells and silencing of circEPSTI1 inhibits cell proliferation.** (**A**) CircEPSTI1 expression in 2 cervical cancer cell lines (CaSki, HeLa) and normal cell line (HcerEpic) examined by qRT-PCR. (**B**) Knockdown efficacy of two distinct si-circEPSTI1 evaluated by RT-qPCR. (**C**) CCK-8 assay in HeLa and CaSki cells transfected with circEPSTI1 siRNAs or control. (**D**) HeLa and CaSki cancer cells were co-transfected with si-control, si- circEPSTI1 mimics. BrdUrd incorporation was evaluated via IHC to assess cell growth after transfection with the si-circEPSTI1 or si-control at 48 hours. **, *P* < 0.01 compared to the negative control group.

The expression level of circEPSTI1 was detected in normal cervical cell line and two cervical cancer cell lines. We found that circEPSTI1 was upregulated in all cervical cancer cell lines, CaSki and HeLa, compared with that in HcerEpic cell line (Figure 1A).

### Silencing of circEPSTI1 inhibits cervical cancer proliferation

We next conducted loss-of-function assays to investigate whether circEPSTI1 was involved in the growth of cervical cancer. RNA interference was utilized to knockdown circEPSTI1 in HeLa and CaSki cervical cancer cell lines to evaluate its potential functions. Two siRNAs were synthesized to knock down circEPSTI1 by targeting the back-splicing region. The expression of circEPSTI1 was decreased after transfected with siRNAs (Figure 1B). Silencing circEPSTI1 suppressed cell proliferation showed by CCK-8 assays (Figure 1C). Furthermore, we used BrdU assay to evaluate proliferative potentiality. BrdU results revealed that thinner nuclear stain dots in the silencing of circEPSTI1 group of both HeLa and CaSki cervical cells than that in the negative control group (Figure 1D).

### CircEPSTI1 affects cervical cancer proliferation *in vivo*

**Figure 2:**
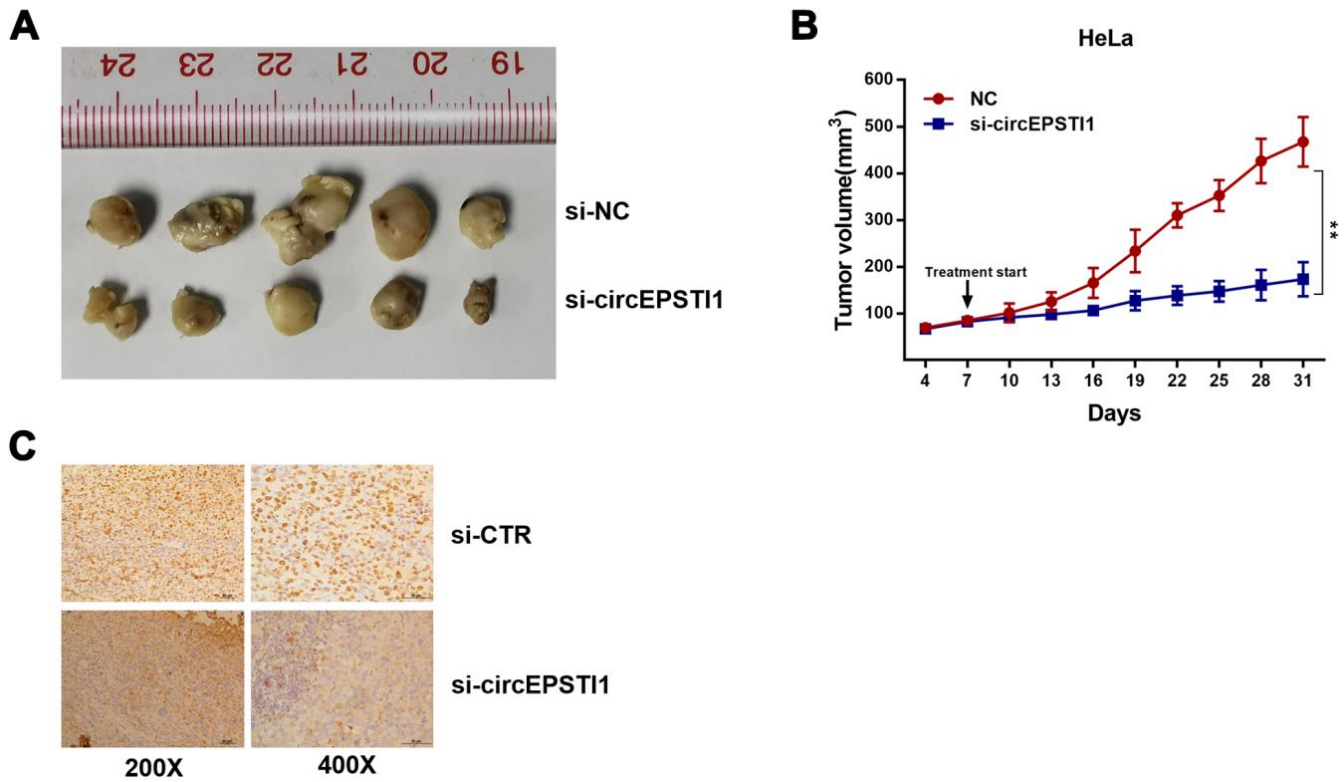
**CircEPSTI1 promotes cervical cancer proliferation *in vivo*.** (**A**) Tumor in the mouse xenograft model. (**B**) The nude mice were subcutaneously injected with HeLa cells and subjected to distinct treatments (i.e., si-cEPSTI1 or scramble). The volume curves of tumors are shown. **, *P* < 0.01 compared to the negative control group. (**C**) The immunohistochemistry analysis was performed for xenograft tumors and the representative images of ki-67 expression are presented. Scale bar = 50 μm.

To figure out the impacts of circEPSTI1 on the proliferation of cervical cancer in mouse assays, HeLa cells were transfected with siRNAs si-cEPSTI1 or scrambled and injected into nude mice subcutaneously. The tumor weight (Figure 2A) and tumor volume (Figure 2B) was decreased in circEPSTI1 knockdown group compared with the scramble control group after intratumoral injection for two weeks. Furthermore, IHC was used to analyze Ki67 protein in tumor mouse xenografts of two groups. In the knockdown group, the Ki67 expression was remarkably decreased in tumor tissues (Figure 2C).

### CircEPSTI1 serves as a miRNA sponge for miR-375, miR-409-3P and miR-515-5p

**Figure 3:**
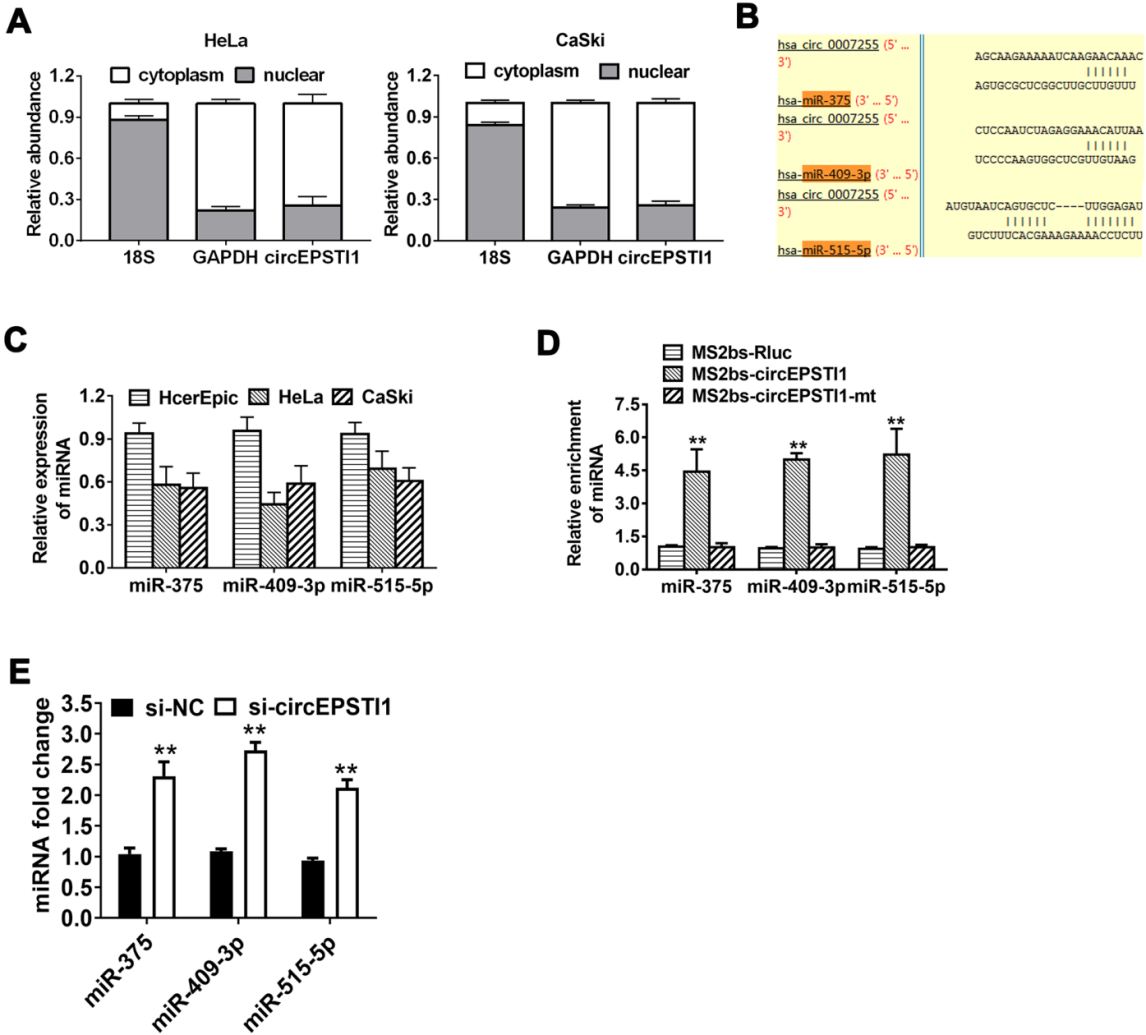
**CircEPSTI1 acts as a sponge of miRNAs for miR-375, miR-409-3P and miR-515-5p.** (**A**) The localization of circEPSTI1 in cell was examined in HeLa cells. (**B**) Schematic diagram of the predicted miR-375, miR-409-3P and miR-515-5p sites in the circEPSTI1. (**C**) QRT-PCR detected the miR-375, miR-409-3P and miR-515-5p levels in cervical cancer cell lines (HcerEpic, HeLa and CaSki). (**D**) MS2-based RIP assay in cervical cancer cells transfected with MS2bs-Rluc vector (control), MS2bs-circEPSTI1 vector or MS2bs-circEPSTI1mt vector. (**E**) Knockdown circEPSTI1 significantly upregulated the expression level of miR-375/409-3P/515-5p. **, *P* < 0.01 compared to the negative control group.

CircEPSTI1 predominantly existed in the cytoplasm indicating that it was able to interact with miRNA (Figure 3A). To explore the mechanism of circEPSTI1 in promoting cancer progression, the Circular RNA Interactome online database was used to assess the potential interaction between circRNA and miRNAs. Among all the candidates, miR-375, miR-409-3P and miR-515-5p were predicted to interact with circEPSTI1 (Figure 3B). In addition, miR-375, miR-409-3P and miR-515-5p were low expression in all the cervical cancer cell lines (Figure 3C). To confirm the interaction between circEPSTI1 and miR-375/409-3P/515-5p, RIP assay was performed. The results revealed that miR-375/409-3P/515-5p was primarily enriched in the MS2bs-circEPSTI1 group (Figure 3D). Knockdown circEPSTI1 significantly upregulated the expression level of miR-375/409-3P/515-5p (Figure 3E). These results indicated that circEPSTI1 increases cervical cancer growth as a sponge for miR-375/409-3P/515-5p.

### SLC7A11 acts as the target of miR-375/409-3P/515-5p and is suppressed by circEPSTI1 knockdown

**Figure 4:**
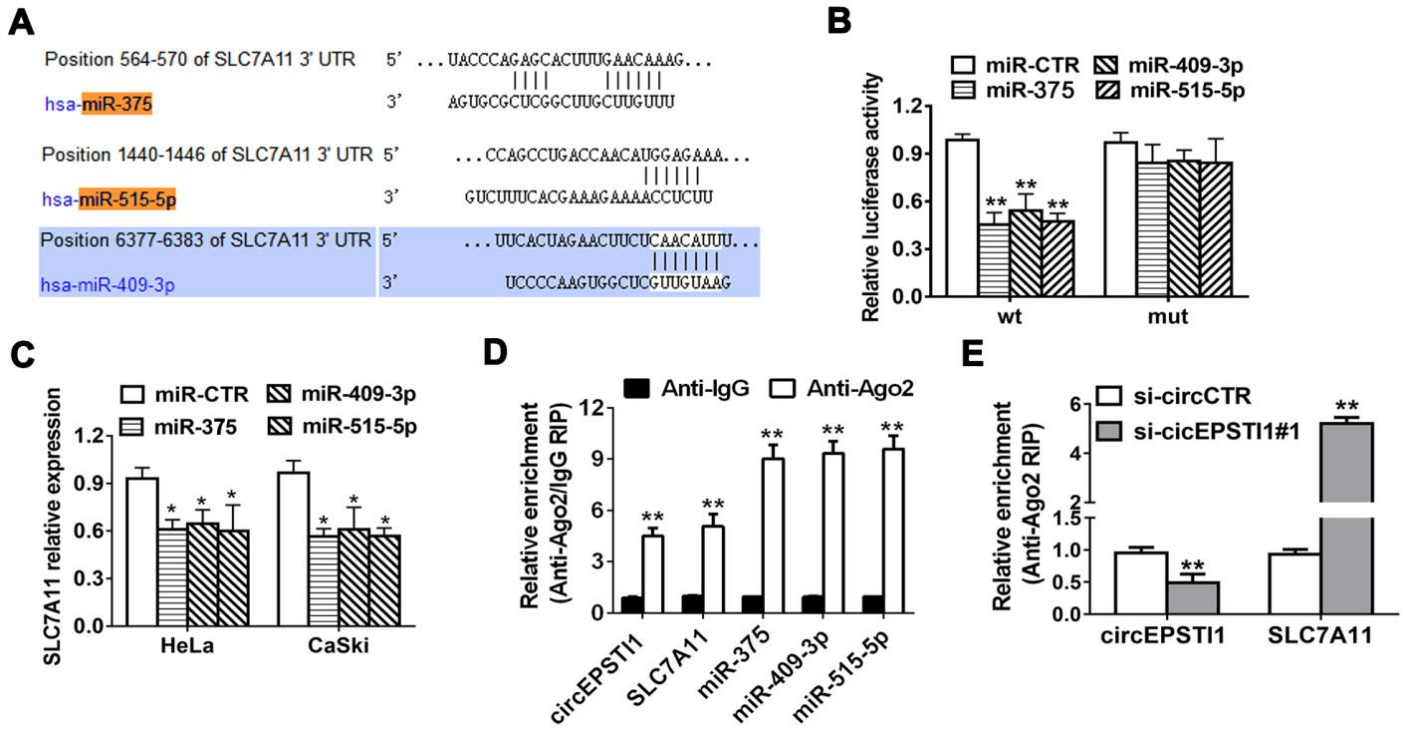
**SLC7A11 acts as the target of miR-375/409-3P/515-5p and is inhibited via circEPSTI1 knockdown.** (**A**) Schematic diagram of the predicted miR-375, miR-409-3P and miR-515-5p sites in the 3′UTR of SLC7A11 mRNA. (**B**) Luciferase assay of cancer cells cotransfected with miR-375, miR-409-3P and miR-515-5p mimics and a luciferase vector containing SLC7A11-3'UTR-wt or mutant constructs with mutated miR-375/409-3P/515-5p binding sites (SLC7A11-3'UTR-mut). (**C**) SLC7A11 expression after transfection with miR-375, miR-409-3p, miR-515-5p mimics and scrambled control detected by QRT-PCR analysis. (**D**) RIP assay revealed that the enrichment of circEPSTI1, SLC7A11 and miR-375, miR-409-3P and miR-515-5p on Ago2 relative IgG. (**E**) A RIP on Ago2 was conducted. *, *P* < 0.05; **, *P* < 0.01 compared to the negative control group.

TargetScan online database algorithm was used to predict potential downstream targets of miR-375/409-3P/515-5p and we identified SLC7A11 as the potential oncogene (Figure 4A). Luciferase reporter assays and RIP assays were performed to determine whether miR-375/409-3P/515-5p could interact with the 3’-UTR of SLC7A11 mRNA. Luciferase reporter assays demonstrated that the activity was remarkably decreased after transfection with miR-375/409-3P/515-5p mimics and wildtype 3’-UTR-SLC7A11 reporter in HeLa cells, while was not significantly different after transfection with mutated type 3’-UTR-SLC7A11 reporter vector (Figure 4B). Moreover, the expression of SLC7A11 decreased by miR-375/409-3P/515-5p mimics in HeLa and CaSki cell lines, indicating that SLC7A11 is downregulated by miR-375/409-3P/515-5p (Figure 4C). Additionally, Ago2/IgG related RIP assay revealed that circEPSTI1, SLC7A11 and miR-375/409-3P/515-5p were all significantly enriched to Ago2 in HeLa cells (Figure 4D). Silencing of circEPSTI1 remarkably increased SLC7A11 enrichment to Ago2 (Figure 4E).

### Silencing of circEPSTI1 induces ferroptosis mediated by SLC7A11

**Figure 5:**
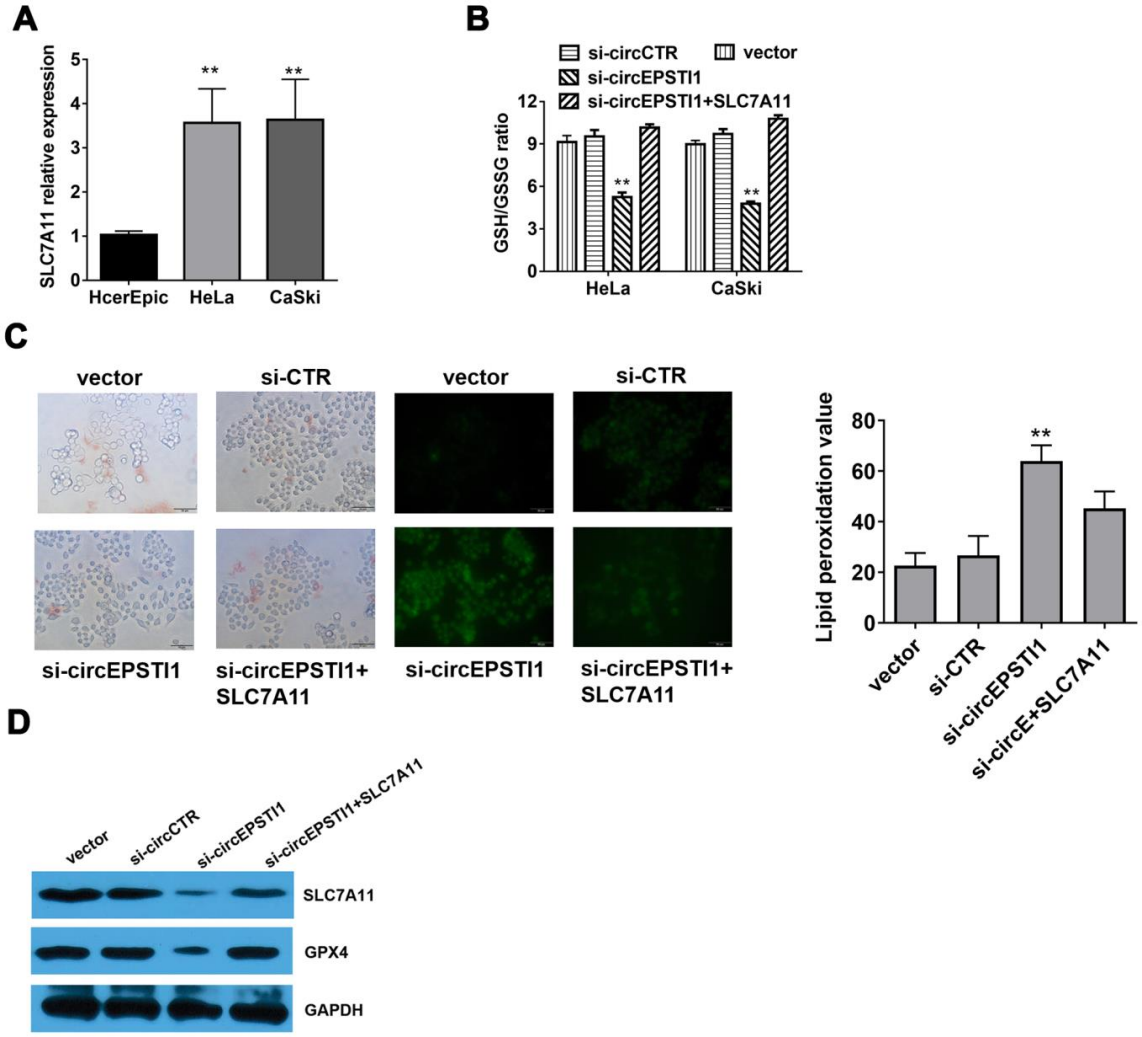
**Silencing of circEPSTI1 induces ferroptosis mediated by SLC7A11.** (**A**) SLC7A11 mRNA level in cervical cancer cell lines were detected by qRT-PCR analysis. (**B**) The glutathione (GSSG) to reduced glutathione (GSH) ratio in HeLa cells was detected by GSH/GSSG assay. (**C**) Immunofluorescence images of HeLa cells treated for si-circEPSTI1, SLC7A11 and vector and stained with liperfluo were shown; scale bar: 50 μm. (**D**)The effect of circEPSTI1 knockdown on SLC7A11 and GPX4 protein level in HeLa cells. **, *P* < 0.01 compared to the negative control group.

Compared with HcerEpic cell line, the expression level of SLC7A11 were upregulated in HeLa and CaSki cell lines (Figure 5A). Quantitative analysis of total glutathione and reduced glutathione in HeLa cells revealed that silencing of circEPSTI1 would reduce the ratio of GSH/GSSG in cervical cancer cells (Figure 5B). The liperfluo-stained cells were observed by laser confocal for the peroxidation accumulation. Detected by cell morphological analysis, the number of HeLa cells decreased significantly, the refractive index decreased, and the polarization showed a long spindle after inhibiting circEPSTI1. However, the tendency was reversed notably by overexpression of SLC7A11. Moreover, silencing of circEPSTI1 significantly increased the accumulation of lipid peroxides on the cell membrane, while overexpression of SLC7A11 significantly reduced lipid peroxide production (Figure 5C). Western blot analysis was used to quantify the markers of ferroptosis inhibitors SLC7A11 and GPX4 in HeLa cells. After silencing of circEPSTI1, the expressions level of SLC7A11 and GPX4 significantly decreased, which were reversed by overexpression of SLC7A11 (Figure 5D).

## DISCUSSION

Recent researches indicate that circRNAs could act as novel monitoring markers and promising treatment targets for tumor therapies. Though circRNAs have been studied for decades, the roles of most circRNAs still remain unknown, apart from some widely known circRNAs, for example, ciRS-7 [14]. Moreover, the roles of circRNAs in cervical cancer are still rarely explored. To the best of our knowledge, this is the first study regarding the relationship between circRNAs and ferroptosis.

CircEPSTI1 is originated from a parental gene, EPSTI1, which is highly involved in epithelial-stromal interactions. Researchers have revealed that the high abnormal expression of EPSTI1 may have a very important role in the invasion and invasion of cancer [27]. circEPSTI1 exerts crucial functions in cancer invasion and metastasis. Chen et al. [18] found that, via the ceRNA mechanism of miR-6809/4753, circEPSTI1 affected the apoptosis and proliferation of breast cancer. Xie et al. [28] demonstrated that circEPSTI1 blocked miR-942 to regulated EPSTI1 expression and affected the progression of ovarian cancer. It’s also reported that the progression of osteosarcoma was affected by circEPSTI1-miR-892b-MCL1 axis [29]. Whether circEPSTI1 exerts a similar or other role in the cervical cancer is unstudied. Therefore, we started to study whether circEPSTI1 plays a vital role in cervical cancer. In the current study, cervical cancer cell lines were found exhibited a higher expression level of circEPSTI1. circEPSTI1 knockdown suppressed the proliferation of cervical cancer cell lines by loss-of-function assays. Furthermore, we demonstrated that silencing of circEPSTI1 reduced the tumor growth in mouse xenograft models of HeLa cell line. Our results confirmed that the circEPSTI1 might serve as an important oncogene and is a potentially promising treatment target for cervical cancer treatment.

The mechanism how circRNAs mainly take effect in cancer is their sponging activity toward miRNA. The main processes of ceRNA mechanism are that the miRNAs tend to be sponged by circRNAs and thier target mRNAs are further freed, constituting the regulatory circRNA/miRNA/mRNA axis. For example, a circular RNA acts as the miR-326 sponge to enhance the progression of cervical cancer via increasing ELK1 expression [30]. Therefore, the ceRNA interaction, could largely involve in the cervical cancer progression mediated by circRNAs. Our results showed that circEPSTI1 took effect through sponging miR-375, miR-409-3P and miR-515-5p to suppress the SLC7A11 expression, which is a putative target of miR-375, miR-409-3P and miR-515-5p. Our study demonstrated that circEPSTI1 regulated SLC7A11 through the ceRNA network consisting of miR-375, miR-409-3P and miR-515-5p.

Ferroptosis is characterized by nonapoptotic and programmed cell death which was initiated after the inactivation of GPX4 and the xc^–^, a cystine and glutamate antiporter system which is consisted of SLC3A2 and SLC7A11, and the following iron-dependent lipid peroxidation [22, 31]. GSH, a reducing substrate of GPX4, plays an important role as a momentous intracellular antioxidant and protect cells from oxidative stress by mitigating the ROS accumulation. Suppression of system xc^−^ could decrease the intracellular level of cystine significantly, repress the metabolism of GSH, and further lead to ferroptosis [22, 32]. Our study showed that silencing of circEPSTI1 reduced GSH to GSSG ratio and the expression of GPX4, finally increasing the lipid peroxides accumulation on the cell membrane. In addition, overexpression of SLC7A11 recovered GSH: GSSG ratio and the expression of GPX4 depleted by silencing of circEPSTI1. In other word, silencing of circEPSTI1 suppressed the expression of SLC7A11 and subsequently inhibited the system xc^–^. Then inhibition of system xc^−^ retarded GSH synthesis and GPX4 cannot convert reduced GSH to GSSG, eventually evolving into lipid peroxidation and inducing ferroptosis. As a result, we identified a novel mechanism that circEPSTI1 could upregulate the SLC7A11 expression and further inhibit ferroptosis.

In summary, our results revealed that circRNAs may also play very important roles in cervical cancer acting as miRNA sponges. We found that circEPSTI1-miR-375/409-3P/515-5p-SLC7A11 axis promotes the cervical cancer cell proliferation through a mechanism involving ceRNA. It is the first time to find that circular RNAs in cervical cancer were associated with ferroptosis. These results indicated that circEPSTI1 may be a treatment target or candidate prognosis markers in cervical cancer. However, more samples are needed for further research.

## MATERIALS AND METHODS

### Cell culture and treatment

All cell lines (CaSki, HeLa, and HcerEpic) were purchased, and cultured following the supplier’s guidance of the ATCC. These cell lines were maintained and passaged for no more than 6 months in laboratory. The detection for mycoplasma infection was performed routinely. Before experiment, the authenticity of all cell lines was verified by DNA fingerprinting.

### Quantitative reverse transcription polymerase chain reaction (qRT-PCR)

The TRIzol reagent was applied for RNA extraction. NE-PER Nuclear and Cytoplasmic Extraction Reagents was used to isolate Cytoplasmic and nuclear RNAs (Thermo Fisher Scientific, MA, USA). A PrimeScript RT reagent kit was used for complementary DNA synthesis (Takara). QRT-PCR was performed with SYBR Premix Ex Taq (Takara Bio, Inc., Dalian, China).

### Transfection

Lipofectamine 3000 (Invitrogen Life Technologies, Carlsbad, CA, USA) was used for cell transfection. miRNA inhibitors or mimics were designed by GeneCopoeia (Rockville). si-circEPSTI1 siRNAs were all synthesized by GenePharma (Shanghai, China).

### Cell counting kit-8 (CCK-8) assay

The CCK-8 assay (Dojindo Laboratories) was used for cell measurement. 1×10^3^ cells per well were cultured in 96-well plates. Ten microliters of CCK-8 solution were added to each well on a certain day. The microtiter plate reader was used to measure the absorbance after 2 hours’ incubation at 37° C.

### Cell proliferation assay

The cell viability assay was performed as previously described [33, 34]. Modulated cancer cells were incubated with culture media (10 nmol/L BrdUrd) for 16 hours and then fixed using cold acetone and methanol (1:1) to evaluate the incorporation of BrdUrd. Immunocytochemistry was subsequently performed.

### RNA binding protein immunoprecipitation (RIP)

Cells were co-transfected with MS2bs-circEPSTI1, MS2bs-circEPSTI1-mt and MS2bs-Rluc using Lipofectamine 3000. RIP was conducted with a Protein Immunoprecipitation Kit (Millipore, MA, USA) after 48 hours. The level of miR-375/409-3P/515-5p was detected. The anti-Ago2 and anti-IgG antibody (Millipore) were applied in the RIP assay for Ago2/IgG. The abundance of circEPSTI1, SLC7A11 and miR-375/409-3P/515-5p were detected after purification.

### Luciferase reporter assay

5×10^3^ HeLa cells per well were prepared in 96-well plates. Plasmids and miR-375/409-3P/515-5p mimics were transfected correspondingly using the Lipofectamine 3000 transfection reagent. The dual-luciferase reporter assay system (Promega, WI, USA) were further applied for Luciferase activity measurement after incubation for 48 hours. All experiments were designed.

### Western blot analysis

Total proteins were extracted, separated by 10% SDS-PAGE and then transferred to PVDF membranes. The membranes were blocked and afterwards incubated with primary antibodies against GPX4 (1:1000; ab231174; Abcam, MA, USA), SLC7A11 (1:500; ab175186, Abcam) and GAPDH (1:3000; ab125247, Abcam). Secondary antibody (CST, MA, USA) was used and quantified by chemiluminescence.

### Reduced glutathione and glutathione (GSH/GSSG) assay

Reduced GSSG (glutathione disulfide) and glutathione (GSH) in cervical cancer cells were detected (Glutathione Detection Kit). All operations are in accordance with the manufacturer's instructions. All experiments were designed and conducted in triplicate.

### Lipid peroxidation measurements and confocal microscopy

The fluorescence-activated cell sorting with Liperfluo staining and confocal microscopy (A1R+Storm, Nikon) were used to lipid peroxidation measurement and detection.

### Mouse xenograft model

Ethical approval was obtained from the Institute Research Ethics Committee of Sun Yat Sen University Cancer Center (SYSUCC), and all the animal procedures were performed in accordance with institutional guidelines. The 4-week-old female BALB/c nude mice were used for constructing xenograft model. HeLa cells (1×10^7^ cells/mL) were injected into the dorsal flanksusing using 1-mL syringes. Then tumor size was measured and mice received intertumoral injection of si-crEPSTI1-1 (40 μL siRNA1 for cicrEPSTI1) and negative control (40 μL negative control) every four days, respectively (5 mice/group). After 28 days, the mice were sacrificed and xenografts were measured. The tumor volumes (mm^3^) were calculated: volume = 0.5×(longest diameter)×(shortest diameter)^2^. The anti-Ki-67 (CST, 1:300) was applied for immunohistochemistry of tumors from each of the mice.

### Statistical analysis

All statistical analyses were conducted with the Prism GraphPad version 6.0 (GraphPad, LaJolla, CA, USA) and SPSS25.0 software package (SPSS, Chicago, IL, USA). Comparisons between groups were analyzed with the Student’s *t* test and one-way ANOVA. Means ± standard deviation (SD) was used to present quantitative data. *P* < 0.05 was considered statistically significant.
